# The Link Between Positive and Negative Parenting Behaviors and Child Inflammation: A Systematic Review

**DOI:** 10.1007/s10578-021-01224-4

**Published:** 2021-08-04

**Authors:** Jacqueline R. O’Brien, Elizabeth C. Loi, Michelle L. Byrne, Maureen Zalewski, Melynda D. Casement

**Affiliations:** 1 Department of Psychology, University of Oregon, 1451 Onyx Street, Eugene, OR 97403

**Keywords:** Parenting, inflammation, cytokines, biomarkers, immune system

## Abstract

Children’s inflammation may be an important link between parenting behaviors and health outcomes. The aims of this systematic review were to: 1) describe associations between parenting behaviors and child inflammatory markers, and 2) evaluate the relevance of existing literature to the review question. Database searches identified 19 studies that included a measure of positive or negative parenting behaviors and a marker of child inflammation, 53% of which measured parental responsiveness/warmth. Greater parental responsiveness/warmth was associated with lower levels of child pro-inflammatory markers in 60% of studies. Across studies, the association between parenting and child inflammation varied as a function of parenting construct, inflammatory measure, and sample characteristics. Studies were highly relevant, with 42% rated 5+ out of 6 for study’s ability to address links between parenting behavior and child inflammation. If future research uncovers causal effects of parenting behaviors on inflammation, parenting interventions could be employed as a preventative tool.

Exposure to stress and toxic environmental factors early in development are routinely shown to confer risk for negative physical and mental health outcomes later in life [1, 2, 3]. Given the strong associations between early stress exposure and later health outcomes, a substantial amount of research is dedicated to understanding the multifaceted pathways by which environmental risk “gets under the skin” to influence children’s development, with an emphasis on biological systems such as the activation of the hypothalamic-pituitary-adrenal (HPA)-axis and autonomic nervous system [e.g., 4, 5, 6]. More recently, the field has begun to examine immune system functioning as an important pathway for this relationship. Inflammation is the biological process through which the body’s immune system responds to pathogens and tissue damage [7]. While acute inflammation is a necessary and adaptive response to stressors, chronic or low-grade systemic inflammation has been found to have an enduring link to later physical disease and psychopathology, including cardiovascular disease, diabetes, cancer, and depression [8, 9, 10, 11, 12, 13].

Inflammatory systems are responsive to a range of psychosocial stressors [14]. In both children and adults, stressors such as low socioeconomic status, social isolation, and chronic stress are associated with increased levels of proinflammatory profiles such as elevated levels of C-reactive protein (CRP), fibrinogen, interleukin-6 (IL-6), and specific gene expression patterns [15, 16, 17]. Increased levels of CRP, IL-6, and other proinflammatory cytokines are also associated with child maltreatment and abuse [18, 19]. There is an emerging area of research that examines whether parenting behaviors that would not meet the threshold for abuse or maltreatment also confer risk for inflammation. Rather than focusing on “extreme” caregiving experiences such as maltreatment, this area emphasizes the importance of normative parenting behaviors on child health outcomes. If variations in normative parenting behaviors are also associated with negative health outcomes in children, this has important clinical implications in the areas of prevention and intervention. The focus of this systematic review is therefore to identify and synthesize existing studies that examine the relationship between normative parenting behaviors and inflammation in children.

## Parenting

Parenting practices, distinct from parenting styles, are specific behaviors that parents engage in to support child development; parenting styles encompass the quality of parent-child interactions that promote a familial emotional climate [20, 21, 22]. This distinction is particularly relevant given that parenting interventions often target specific parenting practices such as increasing responsiveness or limit setting, rather than parenting styles, in order to improve child outcomes [e.g., 23]. One method of organizing parenting practices is to distinguish between positive and negative parenting behaviors. Positive parenting behaviors include, but are not limited to, being warm and responsive, effectively setting limits, providing support and scaffolding, and granting autonomy in developmentally appropriate ways. Positive parenting behaviors are commonly associated with optimal child development, such as the establishment of a secure attachment [24] and socioemotional competence [25]. In contrast, negative parenting behaviors include being harsh, rejecting, withdrawn, inconsistent with limits, and intrusive. Negative parenting behaviors predict problematic childhood developmental outcomes, such as avoidant attachment [24] and higher levels of aggression [26].

Positive and negative parenting have also been shown to impact child biological systems, including autonomic systems that contribute to immune responses. For example, poorer parenting quality, such as lower warmth, is associated with significant differences in children’s HPA-axis functioning [27], while negative parenting has been shown to mediate the relationship between low income and disrupted child cortisol levels [28]. Parenting has also been shown to influence children’s autonomic functioning, such that higher levels of maternal sensitivity are associated with more effective autonomic regulation [29, 30], while higher levels of maternal control are associated with poorer child autonomic regulation [5, 30]. These biological systems are all intricately linked, with glucocorticoid production and stimulation of the vagus nerve directly influencing inflammatory response systems and vice versa [31, 32]. While a meta-analysis shows that child maltreatment is associated with specific inflammatory markers [19], the associations between positive and negative parenting behaviors and inflammatory markers have not yet been synthesized and are the focus of the present review.

## Inflammatory markers

A number of biomarkers within the immune system are involved in the inflammatory process. The immune system commands a series of complex innate and adaptive reactions involving multiple substrates to maintain homeostasis [33]. Inflammatory markers reflect relative levels of immune system activation, with higher concentrations of pro-inflammatory markers reflecting more energy expended by the human immune system to offset biologically- or psychosocially-based perturbations in bodily homeostasis [34]. Pro-inflammatory molecules, such as IL-6 and interferon-γ (IFN-γ), promote the immune system’s efforts to combat threat [18, 33]. IL-6 serves as a progenitor of the acute phase protein CRP, which is also generally understood to support pro-inflammatory conditions [18]. Proteins that trigger anti-inflammatory processes, such as interleukin-10 (IL-10) and interleukin-13 (IL-13), stabilize or suppress the immune system’s activity [18, 35]. The immune system works as a complex and dynamic interplay between pro- and anti-inflammatory processes, such that many anti-inflammatory cytokines also have pro-inflammatory properties [35, 36, 37]. A variety of methods are currently used to assay for inflammatory markers, with serum (blood) and saliva among the most common types of biological samples collected to measure levels of inflammation [e.g., 38, 39]. Further, while circulating levels of inflammatory markers reflect systemic inflammation, stimulated levels of cytokine production indicate the immune system’s capacity to respond to a stressor [40]. While there are benefits to measuring both specimen types, it is important to note that circulating levels of inflammatory markers are often not correlated with stimulated production of corresponding markers [e.g., 41].

Emerging evidence suggests that the immune system may be particularly sensitive to psychosocial input from the environment, and that changes in inflammation may reflect biological embedding of early psychosocial threat. For example, research with adults has demonstrated that a history of maltreatment in childhood is associated with elevated CRP in adulthood [1]. In addition, a growing number of recent studies reveal that inflammation levels in children are associated with the quality of parenting that they experience [e.g., 42], adding to the existing array of biological markers that can be used to characterize the associations between the familial context and children’s physical health. Interactive links between the nascent immune system in childhood and sub-optimal psychosocial conditions within the family environment could shape inflammation later in life [43], and inflammatory markers may be particularly useful in characterizing the long-term physiological legacy of parenting because such indicators can be detected many years following the initial onset of a particular stressor [44]. Understanding the origins of low-grade systemic inflammation in childhood is therefore critical in identifying and potentially intervening upon factors that increase the risk of later morbidity and mortality. This review will focus on parenting behaviors as one component of childhood that may influence child inflammation.

## Aims of the Systematic Review

Much of the research on parenting behaviors and child inflammation has been published within the last six years (i.e., 2014–2020), suggesting that findings in this area of inquiry are emerging simultaneously, which may preclude the opportunity for studies to inform one another. Given the rapid growth in this relatively new field, a synthesis of the findings that have emerged thus far will help ensure that continued investigations into the relationship between normative parenting behaviors and child inflammation proceed in an efficient, cohesive, and cost-effective manner. It is also important to take stock of the ability of existing studies to empirically determine the association between parenting and inflammation, given that effect sizes in other areas of psychoneuroimmunological research, including those pertaining to the association between inflammation and depression, are dependent on variations in study design and methodological rigor [45]. Therefore, the aims of this systematic literature review are to: 1) describe patterns of associations between parenting behaviors (e.g., warmth, parental support) and inflammation markers (e.g., CRP, IL-6), and 2) evaluate the overall relevance of the existing literature to study the association between parenting and child inflammation. While we will use the term “child” throughout the manuscript, given the state of the literature in this area, studies measuring inflammation in adulthood were included to characterize studies that examined the association between parenting behaviors in childhood and levels of inflammation at any age. The use of the term “child” is therefore meant to convey that parenting behaviors refer to the parenting this individual received as a child, rather than the parenting behaviors they themselves may now engage in as an adult.

## Methods

### Article Identification and Screening

**Figure 1.**
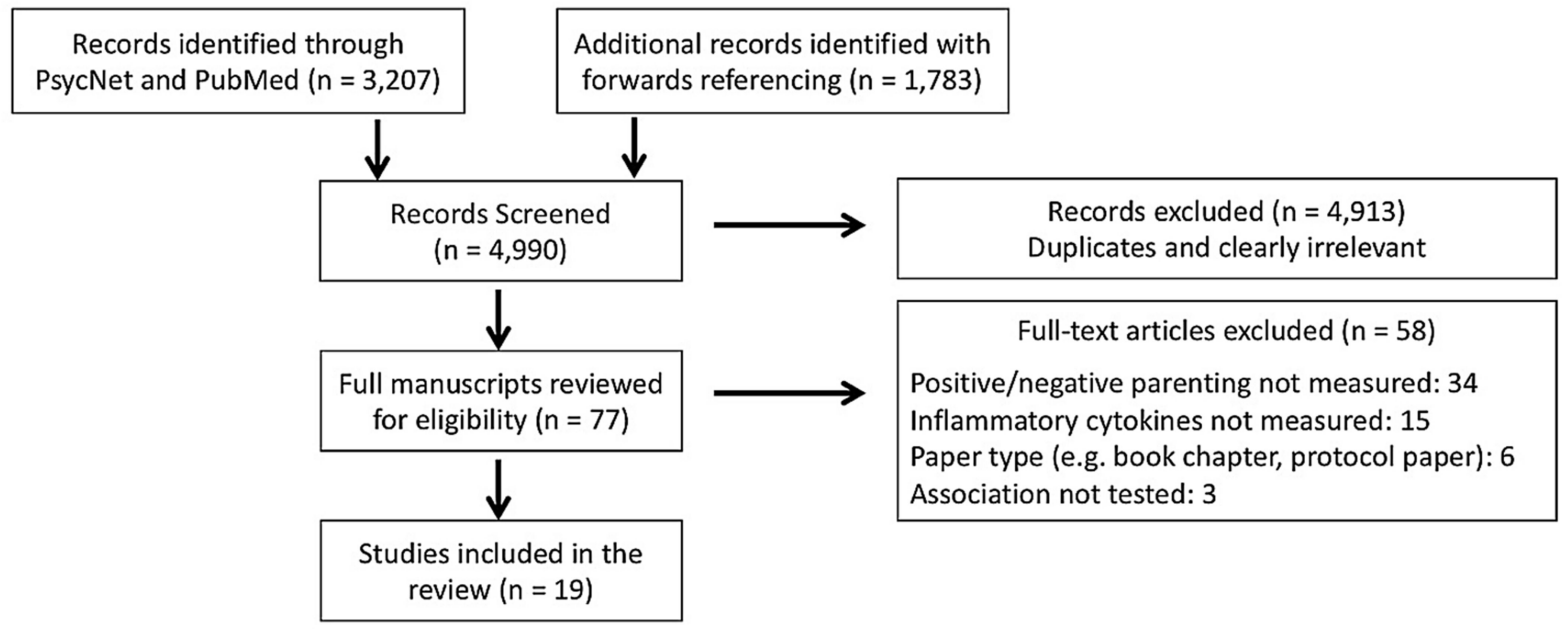
Consort flow diagram for systematic review.

A systematic search was conducted in July 2020 using PRISMA guidelines (see supplementary Table 1) [46], as well as reporting guidelines for synthesis without meta-analysis (SWiM guidelines [87]; see supplementary Table 2). Articles were identified from titles and keywords in PsycNet and PubMed using the following search terms: (parent* OR maternal OR paternal OR attach*) AND (cytokine OR immun* OR inflamm*). These search terms were purposefully broad in order to ensure all potentially eligible studies were captured. Searches were limited to peer-reviewed articles written in English with human subjects. This search yielded a total of 3207 records, and abstracts were screened for relevance. Additional articles (*k* = 1783) were identified using forward referencing by searching Web of Knowledge for articles that cited previously identified articles. In total, 77 full-text articles were identified as potentially meeting eligibility. All identified articles were read in entirety in order to determine eligibility. The full search, identification, screening, and eligibility process is detailed in Figure 1.

### Eligibility

Eligible studies included a measure of both: (1) positive or negative parenting practices experienced during childhood, and (2) a marker of inflammatory profiles. Parenting practices were defined as specific parenting behaviors, including responsiveness and warmth, support, parental involvement, positive or negative/inconsistent discipline, conflict behaviors, behavior monitoring, and use of psychological control. Studies of related constructs such as family context (e.g., general family functioning), parental stress/psychopathology, child maltreatment, or those that measured broader aspects of the parent-child relationship (e.g., attachment), were not included (*k* = 34). Inflammatory markers were defined as the measurement of inflammatory cytokines or acute phase proteins. Studies that measured other aspects of the immune system (e.g., methylation of inflammatory genes) or the presence of child inflammatory disease as the primary outcome were excluded unless specific inflammatory markers were examined (*k* = 15). Qualifying studies included those in which child inflammatory profiles were measured after the age of 18 (*k* = 5) if they were concurrent with retrospective reports of the parenting these individuals received as a child. Studies that did not test the association between parenting and inflammation were excluded (*k* = 3), as were reports that were literature reviews, book chapters, or protocol papers (*k* = 6). In the end, 19 articles met full eligibility criteria for inclusion in this systematic review.

### Data Extraction

The following data were extracted from eligible articles: sample characteristics (size, % male, % non-White, low-income or high adversity sample, clinical population), study design (prospective, retrospective, cross-sectional), and parenting measure (positive or negative; name and description of measure; parenting construct, such as warmth, parental support, parental control, etc.). Parenting construct was coded based on the measure used, rather than how it was framed within the original article, in order to avoid identical parenting measures being coded as different parenting constructs across studies. Data were also extracted regarding inflammatory profile marker and collection technique, age when parenting was measured, age when inflammatory profile was measured, and the time interval between measurement of parenting and inflammatory outcome. The markers CRP, IL-1β, IL-6, IL-8, IFN-γ, and TNF-α were classified as pro-inflammatory markers, while IL-4, IL-5, IL-13, and composite measures were classified as anti- or pro-inflammatory markers depending on the context in which they were measured (e.g., within specific disease models) and how they were derived (e.g., ratio of pro- and anti-inflammatory cytokines) given that many anti-inflammatory cytokines also have pro-inflammatory properties [35, 36]. The direction of the effect between each parenting construct and inflammatory marker was also coded, including any significant interactions. The coding sheet used to support the findings of this review are openly available in Open Science Framework at https://osf.io/znjy9/, reference number DOI 10.17605/OSF.IO/ZNJY9 [47]. Table 1 includes a summary of key study characteristics for each article included in this review, sorted by parenting construct, and then within each parenting construct by significant main effects, significant interactions, and non-significant effects. Table 1 also includes key characteristics of each study (e.g., study design, sample characteristics, sample size, relevance rating) for an informal examination of heterogeneity.

### Systematic Review Relevance Rating

All studies were coded for their ability to answer the specific research questions of this systematic review (i.e., a relevance rating), using an adapted version of a study quality assessment tool developed by Alvares et al. [48]. Three of the authors (M.B., M.C., J.O.) independently rated each study on the following three domains: (1) participants (exclusion/inclusion criteria, generalizability of sample, sample size, inclusion of control condition if applicable); (2) methodology (assessment methods, experimental design, presentation of outcome); and (3) analyses (appropriate analyses conducted, adjusted analyses presented, assessment of confounding factors). Each domain was rated on a scale of 0 (poor/fair), 1 (good), and 2 (excellent). Each study therefore received an overall study relevance rating between 0–6, with higher scores indicating greater confidence in the study’s ability to provide insight on how parenting behaviors are associated with child inflammatory markers. Relevance ratings were used in place of a “quality” rating, as all domains were coded based on their ability to address the specific research questions of this systematic review, rather than their overall scientific rigor. Studies using historically marginalized samples were rated as higher in relevance in the participant domain, even if their samples were less generalizable, because marginalized populations have a higher risk for negative health outcomes [49]. Longitudinal studies that measured both parenting and inflammation at each time point also received higher methodology relevance ratings. Studies that analytically adjusted for factors known to be associated with parenting practices or inflammation (e.g., socioeconomic status, childhood adversity, body mass index) received higher analysis relevance ratings. Discrepancies in overall relevance ratings were resolved through rater discussion and consensus. When consensus could not be reached, the study was given the majority rating. Authors did not assess relevance ratings for any studies they co-authored. See Table 1 for detailed relevance ratings of each study.

### Data Synthesis

Prior to analyzing our review aims, parenting and inflammation data were synthesized and are described here. A total of 19 articles, derived from 15 independent samples, were included in this review. Sample size ranged from 33–756 participants, with a mean sample size of 197 (*SD* = 190). Given that non-independent samples were only included if they examined a unique measure of parenting behaviors or inflammation, summary statistics are derived from the total number of articles rather than the number of independent samples.

#### Measurement of parenting.

Of the 19 articles, 7 (37%) were prospective, 6 (32%) were cross-sectional, 4 (21%) were retrospective, 1 (5%) were intervention studies, and the design of 1 (5%) study could not be determined. There were 18 studies that examined the effects of positive parenting (95%) and 7 studies that examined the effects of negative parenting (37%), with 6 studies measuring both positive and negative parenting (32%). The most common parenting construct measured was responsiveness/warmth (*k* = 10, 53%), followed by parental support (*k* = 7, 37%), conflict behaviors (*k* = 3, 16%), disorganized/insecure attachment (*k* = 3, 16%), negative/inconsistent discipline (*k* = 3, 16%), and then parental involvement/positive discipline and poor behavioral monitoring/high psychological control, which were each examined in two studies (11%). The majority of studies (*k* = 10, 53%) measured parenting in adolescence (age 12–17), followed by adulthood (age 22 or older, *k* = 4, 21%), middle childhood (age 9–11, *k* = 4, 21%), and early adulthood (age 18–21, *k* = 1, 5%). Parenting was not measured before 9 years of age in any of the studies included in this review.

#### Measurement of inflammation.

The most common inflammatory marker measured was CRP (*k* = 12, 63%). Three studies (16%) measured IL-6, while IL-1β, IL-8, IFN-γ, TNF-α, IL-5, and IL-13 were each measured in two studies (11%), and IL-4 was measured once (5%). Four studies (21%) used a composite variable of inflammatory markers. Overall, the median number of inflammatory markers measured per study was 1 (*SD* = 3.12), with a range of 1–14. The majority of studies measured levels of inflammatory markers in serum (*k* = 14, 74%), but studies measuring inflammatory markers in saliva (*k* = 3, 16%) and dried blood spots (*k* = 2, 11%) are noted when applicable. Studies measured either circulating levels of inflammatory markers only (*k* = 13, 68%); stimulated levels of cytokine production only (*k* = 4, 21%); or circulating levels in one marker and stimulated levels in another marker (*k* = 2, 11%). Inflammation was most commonly measured in adolescence (*k* = 8, 42%), followed by adulthood (*k* = 6, 32%), early adulthood (*k* = 4, 21%), and middle childhood (*k* = 1, 8%). Inflammation was not measured before 9 years of age in any of the studies included in this review. While the majority of studies (*k* = 10, 53%) measured parenting and inflammation at the same time point, the average lag between non-concurrent measurements was 8 years (*SD* = 6.95, range = 1–20).

## Results

In order to describe patterns of associations between parenting behaviors and inflammation markers (Aim 1), summaries of the study results are organized by parenting construct. Within each parenting construct, studies are presented in order of significant main effects, significant interactions, and non-significant effects. The framing used to describe the direction of the effect may differ from the original framing presented in their respective articles, in an effort to facilitate comparison of the studies (e.g., framing associations using high, rather than low, levels of parental support). In order to evaluate the state of the current literature regarding the association between parenting behaviors and child inflammation (Aim 2), the final section of the results presents a description of relevance ratings across all parenting constructs in order to capture the strengths and limitations across the three domains (i.e., participants, methodology, analyses) and inform future research in this area.

### Positive Parenting

#### Responsiveness/warmth.

Six studies, from 5 independent samples, found that high levels of responsiveness/warmth were associated with lower levels of inflammation [42, 50, 51, 52, 53, 54]. The first two of these studies found that higher levels of observed parental warmth (both *frequency of behaviors* [42] and *proportions of time* displaying positive behaviors [53]) when children were 12 years of age during a negative problem-solving task were associated with lower levels of salivary CRP three years later [42, 53]. A third study found that maternal responsiveness measured using Electronically Activated Recorders (EARs; devices that collected 50 seconds of sound every 9 minutes over the course of four days) at age 12 was associated with decreased stimulated production of IL-5 and IL-13 but was not associated with stimulated IFN-γ levels [50]. The fourth study found that in a sample of low-income women, higher retrospective reports of maternal warmth at age 31 were associated with lower levels of CRP using dried blood spots [51]. The fifth study found that in a sample with low early-life socioeconomic status, retrospective reports of high maternal warmth at age 33 was associated with lower levels of stimulated IL-6 compared to those who experienced low maternal warmth [54]. However, this same study found no significant difference in circulating CRP levels between the two groups [54].

The sixth study to find a significant association between parental warmth and inflammation found that greater warmth at age 10 was prospectively associated with lower levels of inflammation at age 28 using a composite ratio of 11 pro-inflammatory and 3 anti-inflammatory cytokines in an African American sample [52]. However, this study created a composite measure of parenting that combined parental warmth and hostility/harshness, rather than examining these as independent constructs, thus partly obscuring the ability to determine the relationship between warmth and child inflammation.

Three additional studies did not find a significant association between parental responsiveness/warmth and child inflammation [55, 56, 57]. Two of these studies [55, 56] used retrospective report of parental warmth with concurrent measures of inflammatory markers; large, diverse participant samples (e.g., *N* = 756 Americans balanced by race, gender, and education level); and theoretically informed covariates (e.g., depressive symptoms, socioeconomic risk, body mass index). The third study found no association between adolescent reports of parental warmth and a composite measure of stimulated cytokines, using bivariate correlational analyses [57].

The final study found that adolescent reports of parental warmth was significantly correlated with concurrent measures of stimulated TNF-α, as well as IL-1β that was stimulated with lipopolysaccharide [58]. This association was in the opposite direction than hypothesized, such that higher levels of parental warmth was associated with higher levels of inflammation. In this same study, parental warmth was not associated with circulating levels of CRP and IL-6, stimulated levels of IL-6 and IL-8, or IL-1β in response to cortisol [58].

Overall, these findings provide some evidence that higher levels of parental responsiveness/warmth are associated with lower levels of child inflammation. While four studies did not find any association between parental responsiveness/warmth and child inflammation (or an association in the opposite direction), these studies used either retrospective reports of parenting or bivariate correlational analyses when this association was not the primary research question.

#### Parental support.

Three studies reported that higher levels of parental support were associated with lower levels of inflammation [51, 59, 60], two reported that associations between parental support and inflammation depended on health characteristics of the study samples (e.g., asthma, depressive symptoms) [62, 62], and two reported no relationship between parental support and inflammation [63, 64]. In the three studies with main effects, one study found that higher levels of retrospective maternal support were associated with lower levels of CRP at age 31 in a sample of low-income women [51]. Another study found that higher levels of parental support were concurrently associated with lower levels of stimulated IL-4 production in a clinical sample of adolescents with asthma [59]. One study used a prospective design to assess two measures of parental support (general parental support and parental support in times of need) at ages 12 and 20, and levels of CRP at age 32 in an African American sample [60]. While there was no association between general parental support and CRP, participants who endorsed “almost always” having parental support in times of need at ages 12 and 20 had lower levels of CRP at age 32 [60].

Interaction effects of parental support with other health characteristics were reported by two studies [61, 62]. The first study found that 13-year-old children who reported higher levels of parental support were less resistant to hydrocortisone’s anti-inflammatory effects on stimulated IL-5 and IFN-γ if they had asthma, while this effect was not seen in healthy controls [61]. The second study found that adolescents who reported higher levels of depressive symptoms did not exhibit higher levels of CRP if they also reported experiencing higher levels of parental support [62]. However, adolescents with lower levels of parental support showed increased levels of CRP when they also reported higher levels of depressive symptoms [62].

Two other studies found that parental support measured at age 12 [63] and 18 [64] was not associated with CRP levels at age 20 [63, 64]. These two studies utilized two different questionnaires of parental support at two separate time points but had overlapping low-income African American samples.

Overall, the findings from these seven studies indicate that higher levels of parental support may be associated with decreased child inflammation levels in low-income samples or when additional medical conditions (e.g., asthma, depressive symptoms) are present. However, two published reports with overlapping sets of participants found no relation between parental support and child inflammation.

#### Parental involvement/positive discipline.

One study found a significant association between parental involvement and/or positive discipline and child inflammation. This was an intervention study using a low-income African American sample where 11-year old youth and their parents were randomized to participate in either a 7-week skills-building program designed to increase involved-vigilant parenting, or an active control group of psychoeducation [65]. This intervention targeted consistent, inductive discipline and control, as well as strategies for racial socialization and communication about sex and alcohol use. The results indicated that youth inflammation levels at age 19 were lowest among families whose parents reported higher involved-vigilant behavior after participation in the intervention. Inflammation was measured using a composite of six inflammatory markers: IFN-γ, IL-10, IL-1β, IL-6, IL-8, TNF-ɑ. The second study found that self-reported parental involvement and positive discipline were not associated with child salivary CRP at 9.5 years of age [66]. *Overall, the association between parental involvement and/or positive discipline with child inflammation remains unclear and may be dependent on the inflammatory marker examined or age at which inflammation is measured.*

### Negative Parenting

#### Conflict behaviors.

The first study found that fewer aggressive behaviors by the parent in a negative problem-solving task at age 12 were associated with lower levels of salivary CRP at age 15.5 [42]. However, in this same study, there was no association between parental aggressive behaviors in a positive event planning discussion task and youth CRP at age 15.5 [42]. Using a subset of this sample, the second study found that there was no association between the proportion of time parents displayed aggressive behaviors during a problem-solving task when youth were 12 years old and salivary levels of CRP at age 15.5 [53]. The third study found that in a sample of adolescents with asthma, mother-youth conflict behaviors assessed using Electronically Activated Recorders (EARs) across 4 days were not associated with adolescent stimulated IL-5, IL-13, or stimulated IFN-γ concentrations [50]. *Overall, the association between conflict behaviors and child inflammation remains unclear and may depend on the context in which the conflict behaviors occur, the inflammatory marker being measured, and/or inflammation specimen type (i.e., salivary vs. stimulated).*

#### Poor behavioral monitoring/high psychological control.

The first study found that higher scores on the poor parental monitoring scale of the Alabama Parenting Questionnaire [67] were positively associated with child salivary CRP at 9.5 years-of-age [66]. The second study found that in a sample of 34-year-olds with and without personality disorders, retrospective reports of parental psychological control were positively correlated with IL-6 and CRP concentrations, independent of personality disorder diagnosis [56]. *Together these findings offer preliminary evidence that poor behavioral monitoring and high parental psychological control may be associated with elevated levels of inflammation in childhood and adulthood.*

#### Negative/inconsistent discipline.

The first study found that higher levels of harsh-inconsistent discipline measured at 3 time points in early adolescence (age 11–13) was positively associated with CRP levels in serum at age 19 [68]. The other two studies did not find any association between negative or inconsistent discipline and child inflammation. Specifically, the second study found no association between self-reported negative and inconsistent discipline and child salivary CRP at 9.5 years-old [66]. The final study found that adolescent reports of parental harshness/inconsistency, as well as parental hostility, at age 16 were not correlated with concurrent measures of circulating levels of CRP and IL-6, or stimulated levels of IL-1β, IL-6, IL-8, and TNF-α [58]. It should be noted that it is unclear how harshness/inconsistency and hostility was operationalized in this final study, as this association was not the primary research question. *The association between negative or inconsistent discipline and child inflammation remains unclear and may depend on sample demographics, the age at which inflammation is measured, and/or specimen type.*

### Study Relevance

In order to evaluate the overall ability of the existing literature to empirically examine the association between parenting and child inflammation (Aim 2), study relevance was rated on a scale of 1 (low) to 6 (high). Overall study relevance was high, with the majority of studies receiving a score of 4 (*k* = 6, 32%) or 5 (*k* = 7, 37%). Three studies (15%) received an overall score of 3, 1 study (5%) received a score of 1, and 1 study (5%) received a score of 2. Only one study (5%) received the highest possible score of 6 for overall study relevance.

#### Participants.

Within the participants domain, slightly more than half of the studies (*k* = 10, 53%) received the highest possible score in this domain, while the rest of the studies (*k* = 9, 47%) received a score of 1 out of 2. Every study received at least one point in this domain. The most common reason for diminished relevance in this domain was a small sample size, while studies that received the highest possible score in this domain often examined this research question within a historically marginalized population (e.g., African American participants).

#### Methodology.

The majority of studies (*k* = 13, 68%) received a 1 out of 2 within the methodology domain, while 26% of studies (*k* = 5) received the highest possible score in this domain. Only one study (5%) did not receive any points in this domain. The most common deductions in this domain were due to retrospective reports of parenting, given that it may have weaker associations with biomarkers than prospective measures of parenting [e.g., 69]. Studies that received the highest possible score in this domain often used an intervention design or observed measure of parenting behavior.

#### Analyses.

The majority of studies received the highest possible score in this domain (*k* = 9, 47%), and 37% of the studies (*k* = 7) received a score of 1 out of 2 within the analyses domain. Three studies (16%) received zero points in this domain. The most common deduction in this domain was not sufficiently controlling for confounds that may influence the association between parenting and inflammation (e.g., body mass index, depressive symptoms, socioeconomic risk, early adversity). Either these confounds were not measured, or only correlational analyses without covariates were presented for the analysis of interest.

## Discussion

This systematic review examined the relation between a normative range of parenting behaviors and a discrete set of inflammatory markers (i.e., CRP, IL-6, IL-4, IL-5, IL-13, and IFN-γ) across an array of study designs (i.e., retrospective, cross-sectional, and prospective). In general, the results from this review support the idea that positive and negative parenting behaviors relate to child inflammation. However, the pattern of results varied across parenting constructs and inflammatory markers. In terms of positive parenting, the most consistent findings were that greater parental responsiveness/warmth was linked to lower levels of child inflammation [42, 50, 51, 52, 53, 54], while more general parental support did not have a direct association with child inflammation levels [60, 61, 62, 63, 64]. In regard to negative parenting, poor behavioral monitoring/high psychological control [42, 56] was consistently linked to higher levels of child inflammation. It is important to note that most of the studies in this review focused on prospective measures of inflammation or alternatively, retrospective reports of parenting, in order to offer preliminary findings on how parenting behaviors may be associated with inflammation years later. Taken together, this set of results tentatively suggests that specific dimensions of parenting behaviors may play a role in the development of immune system regulation in children, which has potential consequences for later health.

### Strengths & Limitations of Existing Research

The characterization of the studies identified for inclusion in this review underscores important limitations and strengths in this area of research. First, there were fewer studies that examined negative parenting than examined positive parenting (37% vs 95%, respectively). Studies also predominantly focused on CRP as the inflammatory marker of interest (63%), which limits the connections that can be made across inflammatory markers. Inflammation was also measured using a variety of methods (e.g., serum vs. saliva) and specimen types (e.g., circulating vs. stimulated production). These disparate measurements limit the strength of these conclusions but highlight the need for findings to be consolidated so that the field can move forward in a cohesive manner and pursue the most promising lines of inquiry.

There was also a notable gap in the developmental periods studied in this field, as there were no studies that measured parenting or inflammation before the age of 9 years old. In addition, many of the studies in this review were produced from the same small pool of collaborators and samples, highlighting the need for replication from independent labs and study populations. Although many of the studies included in this review came from a small number of collaborators, the use of covariates across analyses varied greatly. As this field continues to develop, the inclusion of theoretically informed covariates will serve to strengthen the robustness of findings.

One strength of the current state of research at the intersection of parenting and inflammation is that a substantial portion (26%) of these studies have been conducted in high-risk, predominantly African American samples, which have historically been understudied in psychological research. Although the current literature on parenting and inflammation remains limited, the research conducted thus far has been highly relevant, with 42% of studies receiving a score of 5 or higher out of 6 for study relevance to the particular aims of this review. However, it should be noted that 21% of studies used retrospective reports of parenting which has been shown to have weaker associations with biomarkers than prospective measures of parenting [e.g., 69], while 32% of studies measured parenting and inflammation concurrently. Repeated assessments of parenting practices and inflammation would further strengthen conclusions that can be drawn from this work.

### Recommendations for the Future Research

Based on the strengths and limitations of the existing literature in this area, there are five specific recommendations for future research in this field:

1. **Examine associations between parenting behaviors and child inflammation across developmental periods.** Associations between parenting and inflammation have yet to be examined before the age of 9 years old. Parenting behaviors shift as children age, which may have differential effects on the immune system and inflammatory states. For example, longitudinal work by Roberts, Block, and Block [80] on parenting behaviors between children’s ages of 3 and 12 years show that as children get older, parents increasingly emphasize independence and achievement in their children and reduce their degree of physical affection. Examining these associations in early childhood, as well as using repeated assessments across developmental periods and longitudinal research designs, would better elucidate the relationship between parenting behaviors and child inflammation and how these associations change over time.
2. **Select theoretically informed and specific parenting behaviors that are consistent with the aims of the research study.** Positive and negative parenting behaviors may have distinct influences on child inflammation and should therefore be treated as distinct constructs. This review offered more evidence on the association between positive parenting behaviors and child inflammation than negative parenting behaviors, and specifically that warmth/responsiveness was more consistently associated with lower levels of child pro-inflammatory markers than parental support. However, more research is needed on the association between negative parenting behaviors and child inflammation. When conducting research in this area, researchers should carefully choose whether to use a risk or protective lens for their work, as this will inform whether positive or negative parenting behaviors should be examined. Using a risk framework would focus on how negative parenting behaviors place children at risk for higher levels of inflammation while a protective framework would examine how positive parenting behaviors may mitigate the risk of child inflammation within the context of other factors of adversity (e.g., low socioeconomic status). In order to inform areas of intervention, researchers should also consider measuring specific parenting behaviors that have been proven to respond to parenting interventions.
3. **Choose theoretically informed inflammatory markers that are collected using methods appropriate for the aims of the research study.**
  - *Choosing appropriate inflammatory markers.* Evidence suggests that different inflammatory markers may be sensitive to certain kinds of stress and not others. It is therefore important to consider the type of adversity being examined when choosing which inflammatory marker to measure. For example, physical abuse early in life has been found to be unrelated to CRP, and only marginally related to IL-6 [19], while parental absence has been linked to CRP levels [19]. Taking composite measures of early adversity represents another approach to the operationalization of childhood stress. For example, Carpenter and colleagues [79] found that adults who had experienced early life stress as assessed on a multi-domain questionnaire exhibited stronger IL-6 reactivity to a laboratory stress paradigm. Moreover, investigations of inflammatory states often focus on levels of pro-inflammatory and anti-inflammatory markers. While it may be informative to examine concentrations of individual classes of inflammatory markers, it may also be useful to scrutinize the ratio of pro-inflammatory and anti-inflammatory markers, as this may be a driver of neurodevelopmental deficits [37]. The immune system develops and functions along with other systems in the body, not as an isolated entity. In order to capture a holistic picture of how the immune system may be involved in manifesting the effects of parenting on later mental and physical health outcomes, it will also be important to measure inflammatory markers in conjunction with additional measures of physiological functioning.
  - *Choosing an appropriate method of collection*. As a growing number of studies within the field of developmental science measure inflammatory markers, the availability of different data collection methods affords investigators a degree of flexibility in designing their assessment strategies to align with project constraints. While the most common method of measurement for inflammatory markers has been serum samples [e.g., 68, 76], alternative assay techniques permit the detection of inflammation through saliva [e.g., 74] and dried blood spots [e.g., 77]. Some recent evidence suggests that saliva may even prove to be a superior medium relative to serum for quantifying inflammatory states [78]. Researchers should also use the specimen type that corresponds with the aims of their study, as circulating and stimulated production of inflammatory markers may reflect different aspects of immune functioning [40]. In addition, the most common method of immunoassay relies on enzymes in order to measure the concentration of inflammatory markers (i.e., enzyme-linked immunosorbent assays; ELISAs). This is a well-validated and relatively inexpensive technique, but it unfortunately limits the number of inflammatory markers that can be measured within one sample. Other techniques, such as luminescence immunoassays, which use light to measure biomarker concentrations, allow for more inflammatory markers to be evaluated within a single sample. As these techniques become more popular and affordable, studies will be able to expand the scope of their investigations to include a wider array of inflammatory markers.
4. **Continue to replicate these findings across independent labs and study populations, including historically marginalized samples.** To ensure generalizability of findings, it will be important for these associations to be studied by new research collaborators using independent samples. These investigations should also continue to be replicated in historically marginalized samples, including ethnically diverse and low-income samples, to ensure these results can be generalized to other groups. Given the known association between early adversity and inflammation [e.g., 15], as well as higher levels of inflammation due to experiences of marginalization and discrimination [e.g., 81], it is even more vital that research in this area continues to be replicated in historically marginalized samples and populations with elevated risk for negative health outcomes.
5. **Systematic consideration of relevant covariates during analyses.** Future work would benefit from including systematic consideration and reporting of covariates in analyses, particularly given that study design and methodological rigor has also been found to moderate effects in other areas of psychoneuroimmunological research [45, 48]. Relevant covariates include factors known to be associated with parenting practices (e.g., cultural factors) and inflammation (e.g., body mass index, medication use). Future research should also consider confounds related to social determinants of health (e.g., adverse childhood experiences, socioeconomic status) in order to evaluate whether parenting behaviors in particular are influencing inflammation levels.

### Strengths & Limitations of the Current Review

This review was the first to thoroughly synthesize the growing field of research on normative parenting behaviors and child inflammation. The majority of studies (79%) included in this review were published within the last six years (i.e., 2014 – 2020), which exemplifies the need for these findings to be comprehensively integrated to ensure that this field of research is moving forward in a cohesive manner. Due to the range of parenting constructs and inflammatory markers measured within this review, however, a meta-analysis could not be conducted. While this further highlighted the need for a systematic review in this area, effect sizes could not be weighted or compared across studies, and a statistical approach could not be used to measure publication bias. It is notable that the smaller samples in this review had more significant effects than the studies with larger samples, which suggests that publication bias may be relevant. Finally, while it is theorized that parenting impacts child health outcomes through its influence on the immune system [e.g., 43], it is also important to note that this review cannot offer causal evidence for this pathway.

### Clinical Implications

If future research continues to find associations between parenting behaviors and child inflammation, parenting interventions may be an important tool for prevention and intervention given that parenting behaviors are highly responsive to intervention in both clinical and non-clinical populations [85]. Researchers should continue to include child inflammation as an important outcome variable that may link early environmental experiences with later health outcomes and begin to explore additional factors that could serve to buffer against or amplify the ties between parenting and inflammation. A growing number of studies are translating empirical knowledge into interventions aimed at disrupting the course of chronic inflammation. For example, Pace and colleagues [74] evaluated whether Cognitively Based Compassion Training (CBCT) [75] could ameliorate CRP levels among adolescents who were part of the foster care system. While their results indicated that CBCT did not lead to a significant improvement in CRP levels among members of the treatment group relative to the waitlist control group, much more can be learned about the effects of this and other interventions on immune functioning [74]. In order to capture the full range of change that occurs in parenting behaviors from early childhood through adolescence, it is also critical that future investigations adopt a longitudinal approach to examining the links between parenting and inflammation. The results of such studies would not only aid in addressing the question of what types of interventions would be most appropriate for targeting parenting and inflammation, but also help identify for whom and at what age discrete interventions are most effective.

### Summary

This review found that positive and negative parenting behaviors are associated with child inflammation, but these effects varied as a function of parenting construct, inflammatory measure, and sample characteristics. In general, higher levels of positive parenting were associated with lower levels of child inflammation, while higher levels of negative parenting were linked to elevated levels of child inflammation. However, fewer studies examined the relationship between negative parenting and child inflammation than positive parenting and child inflammation. Broader interest in the link between parenting and inflammation is warranted given that much of the work in this area is limited to a small number of investigators and study samples. Links between parenting and inflammation also have yet to be thoroughly examined in children before the age of 9 years old, despite the changing nature of parent-child dynamics during this period of development [80]. Given that parenting is highly responsive to intervention [85] and that systemic low-grade inflammation is consistently linked to negative mental and physical health outcomes [8, 86], this area of research has important clinical implications that warrant further study.

As the body of empirical knowledge at the intersection of parenting and inflammation grows, we as a field are confronted with the question of how to innovate. As we continue to understand how to interpret absolute and relative levels of different inflammatory markers, it is critical to consider how we may use this knowledge to create positive change in children exposed to childrearing practices that may undermine their development. Examination of the immune system and inflammatory processes offers opportunities to gain novel insight into the science of parenting and future health outcomes.

## Supplementary Material

1737169_S1

1737169_S2

## Figures and Tables

**Table 1.** Summary of Findings and Characteristics of Studies Included in Review

	PRO							PRO/ANTI									

	CRP	IL-1β	IL-6	IL-4	IL-8	IFN-γ	TNF-α	IL-5	IL-13	Composite	N	Sample	Design	Type	Age at IV	Age at DV	Rating
**POSITIVE PARENTING**																	
**Responsiveness/Warmth**																	
Byrne et al., 2017	↓										61	-^†^	PRO	SAL	12	15.5	5
Nelson et al., 2017	↓										33	-^†^	PRO	SAL	12.3	15.5	3
Tobin et al., 2015						-		↓	↓		43	AS	CS	SER	12.6	12.6	4
Lyons et al., 2019	↓										52	SES	RETRO	DBS	30.8	30.8	4
Chen et al., 2011	-		↓								53	SES	RETRO	SER	33	33	3
Beach et al., 2017b										↓	413	AA	PRO	SER	10.5	28	4
Carroll et al., 2013										-	756	-	RETRO	SER	40	40	5
Fanning et al., 2015	-		-								134	MH	RETRO	SER	34.3	34.3	5
Manczak et al., 2018										-	123	-	-	SER	14.6	15.6	2
Human et al., 2016	-	↑	-		-		↑				95	-	CS	SER	15.9	15.9	1
**Parental Support**																	
Lyons et al., 2019	↓										52	SES	RETRO	DBS	30.8	30.8	4
Chen et al., 2007				↓							78	AS	CS	SER	12.8	12.8	3
Jones et al., 2017	↓										59	AA	PRO	SER	12/20	31.9	4
Jones et al., 2017	-										59	AA	PRO	SER	12/20	31.9	4
Miller et al., 2009						-		-	-		133	AS	CS	SER	13.3	13.3	5
Guan et al., 2016	-										316	-	CS	DBS	16.4	16.4	5
Beach et al., 2017a	-										382	AA^‡^	PRO	SER	12.4	20.5	4
Brody et al., 2014a	-										331	AA^‡^	PRO	SER	18.0	20.2	5
**Parental Involvement/ Positive Discipline**																	
Miller et al., 2014										↓	272	AA^‡^	INT	SER	11	19	6
Byrne et al., 2016	-										102	-	CS	SAL	9.5	9.5	5

**NEGATIVE PARENTING**																	
**Conflict Behaviors**																	
Byrne et al., 2017	↑										61	-^†^	PRO	SAL	12	15.5	5
Nelson et al., 2017	-										33	-^†^	PRO	SAL	12.3	15.5	3
Tobin et al., 2015						-		-	-		43	AS	CS	SER	12.6	12.6	4
**Poor Behavioral Monitoring/High Psychological Control**																	
Byrne et al., 2016	↑										102	-	CS	SAL	9.5	9.5	5
Fanning et al., 2015	↑		↑								134	MH	RETRO	SER	34.3	34.3	5
**Negative/Inconsistent Discipline**																	
Brody et al., 2014b	↑										368	AA^‡^	PRO	SER	11.2	19.2	4
Byrne et al., 2016	-										102	-	CS	SAL	9.5	9.5	5
Human et al., 2016	-	-	-		-		-				95	-	CS	SER	15.9	15.9	1
Human et al., 2016	-	-	-		-		-				95	-	CS	SER	15.9	15.9	1

*Note*. Studies measuring multiple parenting constructs are listed more than once. ↓= significant negative effect; ↑ = significant positive effect; - = no significant effect; CRP= C-reactive protein; IL= Interleukin; N= Sample size; IV= Independent variable (parenting); DV= Dependent variable (inflammation); MH= Mental health disorder; AS= Asthma; SES= Low socioeconomic status; AA= African American; MAL= History of child protective services; RETRO= Retrospective; PRO= Prospective; CS= Cross-sectional; INT= Intervention; SAL= salivary sample; SER= serum sample; DBS= dried blood spots

† = Non-independent sample

‡ = Non-independent sample
